# Formation of the Submicron Oxidative LIPSS on Thin Titanium Films During Nanosecond Laser Recording

**DOI:** 10.3390/nano10112161

**Published:** 2020-10-29

**Authors:** Dmitry A. Sinev, Daria S. Yuzhakova, Mikhail K. Moskvin, Vadim P. Veiko

**Affiliations:** 1 Faculty of Laser Photonics and Optoelectronics, ITMO University, 49 Kronverksky Pr., bldg. A, 197101 St. Petersburg, Russia; dsyuzhakova@itmo.ru (D.S.Y.); mkmoskvin@itmo.ru (M.K.M.); vadim.veiko@mail.ru (V.P.V.); 2 Institute of Automation and Electrometry of the Siberian Branch of the Russian Academy of Sciences (IA&E SB RAS), 1 Academician Koptyug ave., 630090 Novosibirsk, Russia

**Keywords:** LIPSS, LSFL, laser thermochemical recording, titanium films, laser-induced oxidation

## Abstract

Laser-induced periodic surface structures (LIPSSs) spontaneously appearing on the laser-treated (melted or evaporated) surfaces of bulk solid materials seem to be a well-studied phenomenon. Peculiarities of oxidative mechanisms of LIPSS formation on thin films though are far less clear. In this work, the appearance of oxidative LIPSSs on thin titanium films was demonstrated under the action of commercially available nanosecond-pulsed Yb-fiber laser. The temperature and energy regimes favoring their formation were revealed, and their geometric characteristics were determined. The period of these LIPSSs was found to be about 0.7 *λ*, while the modulation depth varied between 70 and 110 nm, with high stability and reproducibility. It was shown that LIPSS orientation is rather easily manageable in the regimes of our interest, which could provide a way of controlling their properties.

## 1. Introduction

The formation of laser-induced periodic surface structures (LIPSSs), which was observed on a wide range of materials under the action of laser radiation, is a well-known phenomenon that attracts both fundamental and applied attention in modern photonics. LIPSS submicron reliefs are used to control optical [1,2,3,4], mechanical [5], chemical [6,7,8], and other properties of functional surfaces. A significant number of studies have been devoted to the study of LIPSSs appearing under pico- and femtosecond laser exposure [4,9,10,11], often localized in small areas of the irradiated region. LIPSS uniformity and long-range order are also usually disturbed [11] by the simultaneous initiation from multiple independent seed locations [12]. In general, there are two types of LIPSSs: structures with a period comparable to the radiation wavelength *λ* (low-spatial-frequency LIPSS (LSFLs)) and small-scale structures, the period of which is much less than *λ* (high-spatial-frequency LIPSSs (HSFLs)) [13]. In [14], LSFLs were obtained on a Cr film under femtosecond exposure to Yb:KGW laser radiation (wavelength 1026 nm, pulse duration 232 fs) in a nondestructive regime. Those structures, however, significantly protruded above the surface of the initial film, which according to the authors indicated the significant oxidation of the irradiated metal in the areas of the protrusions.

Despite the fact that the mechanisms, controllability, and reproducibility of the LIPSS appearance, presumably, should not significantly depend on the duration of laser exposure, studies of LIPSS formation under nanosecond pulses [15,16,17] are uncommon. This is due to the narrow range of recording parameters and the sensitivity of LIPSS characteristics to the physical mechanisms of their formation, which are easier to identify under a short exposure. The physical mechanisms of LIPSS formation have been discussed in many works, e.g., [13,18,19,20], mainly, however, for bulk metals irradiated by ultrashort pulses with energy densities above the ablation threshold. The mechanisms of LIPSS formation on thin metal films in nondestructive regimes have been studied less thoroughly, although these films are widely used for different applications—from diffraction optical components recording to color laser marking [21]—which increases the interest in the phenomena (oxidation, melting, ablation, etc.) of their laser treatment. The formation of oxidative LIPSSs in [12,14,22] can be represented as the result of the interference of the incident laser radiation, with that scattered by the initial film roughness. Interference leads to the formation of a periodic absorption profile and appropriate modulation of the temperature distribution along the surface. At the maxima of the temperature profile, oxidation occurs more rapidly due to the nonlinear nature of the oxidation process [23], which completes a positive feedback. In addition, the protruding oxide lines and dots can serve as cylindrical or spherical convex nano- or microlenses that redistribute and concentrate the energy of the next laser pulses closer to the lower oxide–metal interface, thereby exacerbating temperature modulation and promoting the formation of oxidative LIPSSs.

In [12], it was suggested that the usage of ultrashort pulses is necessary for LIPSS formation, in order to ensure that heat diffusion does not smear out the nanometer-scale localization of the deposited laser energy. In this work, we present experimental results showing that regular oxidative LIPSSs, which have not been previously observed in the range of low-temperature (below the melting point) processes on thin titanium films, can be obtained under the action of nanosecond laser pulses. Recording, in this case, was conducted using the method of direct laser thermochemical writing on thin metal films (for instance, on Ti [21,23,24,25,26,27], Sn [28], V [29], Zr [29], etc.), in which the image is created by direct oxidation of the original film under local laser heating until the formation of a contrasting transparent oxide layer (for further information about this method, see [23]). The formation of LIPSSs accompanies the oxidation of the initial film and creates an additional relief over the recorded image.

Since the main field of application of the technology of laser thermochemical recording on thin metal films is recording diffractive optical elements [23,30], self-organization of LIPSSs in the irradiated region can be regarded either as a defect (leading to distortion of the wavefront of radiation interacting with the diffractive element) or, on the contrary, as an advantage (as it makes it easier to form structures with a period on the order of the wavelength), with the prospects of being used for direct writing of photonic crystals, specific diffractive elements, etc.

## 2. Materials and Methods

**Figure 1:**
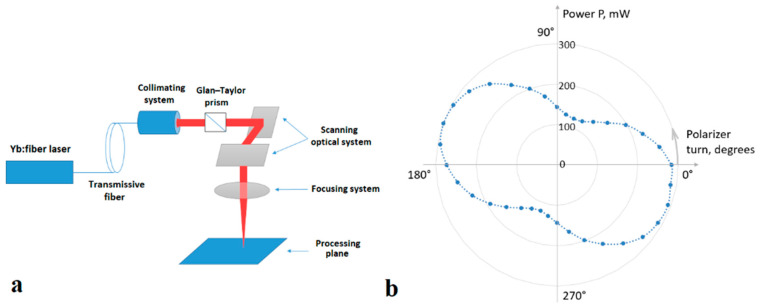
(**a**) Experimental setup diagram; (**b**) results of measuring the power of laser radiation after the optical system depending on the rotation angle of the polarizer (Glan–Taylor prism).

To obtain LIPSSs, in the present work, we used titanium films with the initial transmission in the visible range of about 10% (thickness on the order of 10 nm) on a BK7 glass substrate with a thickness of 2.85 mm. Yb-fiber laser (*λ* ≈ 1.07 μm) with random (partial) polarization and Gaussian spatial distribution of radiation was used as a radiation source, as part of the MiniMarker laser processing complex (Laser Center Ltd., St. Petersburg, Russia) (Figure 1a,b, for technical parameters see [31]).

For determining the regimes suitable for obtaining LIPSSs, the following parameters were varied: pulse duration *τ* (from 4 to 50 ns), average power *P_av_* (from 20 to 400 mW), and pulse repetition rate *f* (from 10 to 100 kHz). Ti films were irradiated with a scanning focused laser beam; the scanning speed *V* remained constant and equal to 0.1 mm/s. With an increase in the scanning speed, LIPSS formation was not registered, possibly due to a decrease in the number of pulses per unit area, down to values at which the oxide did not have enough time to form. Laser radiation was initially partially polarized, and in some experiments a Glan–Taylor prism was introduced into the optical scheme, changing the polarization to mostly linear. The diameter of a Gaussian laser beam on the surface of the film *d* was about 80 μm, and the irradiation of the film was carried out perpendicularly to the surface. Note that under the studied conditions, the pulse overlap coefficient calculated by formula [32]

(1) k=(1−Vfd)×100%

exceeded 99%, and the distance between the centers of neighboring irradiated zones under the action of successive pulses was on the order of 1–2 nm, which, as will be seen below, does not coincide with the period of emerging LIPSSs. Thus, the LSFLs obtained in this work were indeed formed by the mechanism of LIPSS formation and do not represent the edges of the heat-affected zone (HAZ) from neighboring pulses or other similar structures. The obtained structures were examined using the Carl Zeiss Axio Imager A1.m optical microscope (Carl Zeiss Microscopy GmbH, Munich, Germany) and the AFM NT-MDT Nanoeducator (LLC “NT-MDT”, Moscow, Russia). 2D-FFT analysis of optical micrographs was carried out using a homemade image processing program written in Python. The FLIR Titanium 520M (FLIR Systems, Inc., Wilsonville, OR, USA) thermal imaging camera was used to estimate the temperature on the film surface during irradiation, and the power meter Gentec-EO SOLO2 (Gentec Electro-Optics, Inc., Quebec, Canada) was used to measure the average laser power.

## 3. Results and Discussion

**Figure 2:**
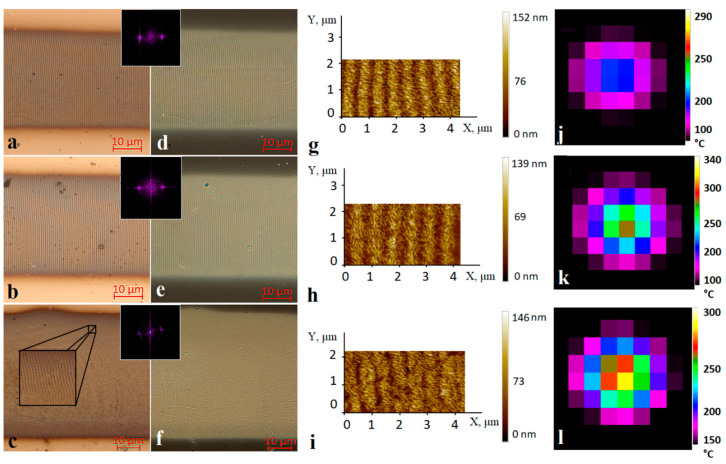
Optical micrographs of the recorded oxide microstructures in reflected (**a**–**c**) and transmitted (**d**–**f**) light, AFM images of surface fragments containing laser-induced periodic surface structures (LIPSSs) (**g**–**i**), and the corresponding thermal images (**j**–**l**). Insets show 2D-FFT spectra of fragments of the corresponding images (spatial frequencies up to 2 μm^–1^). The scanning direction by the laser beam in the images (**a**–**f**) is from left to right. All recordings were conducted with initial partially polarized laser beam. Recording regimes: (**a**,**d**,**g**,**j**) *τ* = 4 ns, *P_av_* = 90 mW, *f* = 50 kHz, *ε* = 20 mJ/cm^2^; (**b**,**e**,**h**,**k**) *τ* = 4 ns, *P_av_* = 140 mW, *f* = 100 kHz, *ε* = 24 mJ/cm^2^; (**c**,**f**,**i**,**l**) *τ* = 8 ns, *P_av_* = 160 mW, *f* = 50 kHz, *ε* = 44 mJ/cm^2^.

Figure 2 shows optical and AFM micrographs of structures recorded at pulse durations *τ* from 4 to 8 ns and fluences *ε* from 20 to 44 mJ/cm^2^, as well as the corresponding measured temperature distributions. There was no dependence of the LIPSS period on the laser energy parameters to be found; in all the cases studied, the LIPSS period was about 0.72 ± 0.02 µm, which corresponds to the values acquired for the films of similar thickness in [12]. The AFM results (Figure 2g–i) show that the LIPSS amplitude also averages at 70–110 nm without clear dependence on the exposure parameters, which indicates the reproducibility and stability of such structures.

Based on the data obtained, it was determined that the optimal value of the average energy density for the appearance of LIPSSs in the oxidative mode lies in the range from 20 to 60 mJ/cm^2^, while the experimentally determined average temperature on the surface does not exceed 350 °C, based on thermal imaging pictures (Figure 2j–l). The peak temperature value reached in the center of the irradiated region on the film at the end of a single pulse was estimated by the following formula [32]:

(2) $$T_{1}=\frac{qA_{1}\tau }{\rho_{1}c_{1}h\left(1+\frac{\sqrt{\pi }}{2\psi }\right)}+T_{in},$$

where *q* is the intensity of laser radiation; *A* is the absorbance coefficient; τ is the pulse duration; *ρ* is the density; *c* is the thermal capacity; *a* is the thermal diffusivity of the materials; $\psi =\frac{\rho_{1}c_{1}h}{\rho_{2}c_{2}\sqrt{a_{2}t}}$ is the coefficient, defining the amount of heat transfer to the substrate; index “1” is related to the metal film; index “2” is related to the oxide layer; and $T_{in}$ is the initial temperature (≈20 °C). The values of the physical and optical parameters of the materials used in the calculations are given in Table 1.

Since titanium oxides are substantially transparent in the visible and near-IR ranges, the estimates obtained by formula (2) using the absorbance of the initial metal film *A_1_* show the upper limit of possible temperature values. Heat accumulation effects under the studied conditions are negligible due to heat dissipation into the substrate during the time between pulses. Model estimations of peak temperature values (as well as experimental average evaluations (Figure 2i–l)) show numbers significantly lower than titanium melting point (1660 °C [33]) and, to be precise, range from 470 to 1065 °C for pulse durations of 4–14 ns within the experimentally determined fluence regimes of oxidation with the appearance of clearly defined LIPSSs. Interference modulation during LIPSS formation may have increased the peak temperature values in the maxima of the interference pattern, but microscopy results (Figure 2a–i) show no evidence of melting, or other signs of thermally-induced aberrations, which makes plausible the nonablative, oxidative nature of the LIPSS formation process.

Although direct comparison of our results with those from other sources is difficult due to differences in selected materials or exposure durations, LIPSSs with similar quality were formed under laser irradiation of the same wavelength, and average powers of the same order, in the benchmark article [12]. However, the laser spot coverage area size in our case is 7 times larger (80 µm vs. 12 µm), and scanning speed is far higher (100 µm/s vs. 2–8 µm/s), which results in much more efficient LIPSS recording, all without violation of long-range order. In addition, the usage of a commercially available nanosecond-pulsed Yb-fiber laser system instead of a femtosecond laser system is economically beneficial.

**Figure 3:**
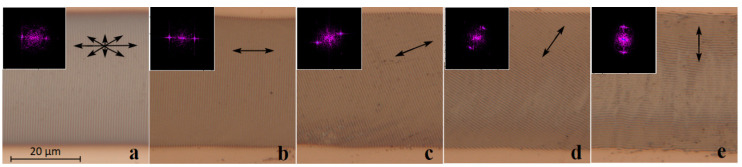
Optical micrographs of LIPSSs recorded in the same energy regime: *τ* = 4 ns, *P_av_* = 90 mW, *f* = 50 kHz, *ε* = 20 mJ/cm^2^. (**a**) Original partially polarized laser beam. (**b**–**e**) Linearly polarized radiation, where double-sided arrows indicate the direction of the polarization plane (at 0, 30, 60, and 90° to the scanning direction respectively). Insets show 2D-FFT spectra of fragments of the corresponding images (spatial frequencies up to 2 μm^−1^).

The well-known result, according to which the orientation axis of the LSFLs is defined by the polarization axis of laser radiation [13], was experimentally confirmed using a Glan–Taylor prism. The original partially polarized laser beam (Figure 3a) was converted using a prism to a linearly polarized one, and the angle of the LIPSS orientation corresponded to the rotation angle of the prism polarization axis. Based on the results of 2D-FFT analysis for structures formed under the action of linearly polarized radiation (Figure 3b–e), the period was found to be the same, i.e., 0.72 ± 0.02 μm. Separately, it was noted that the scanning direction (left-to-right, top-down) of the linearly polarized laser beam does not affect the rotation angle of the LIPSSs.

**Figure 4:**
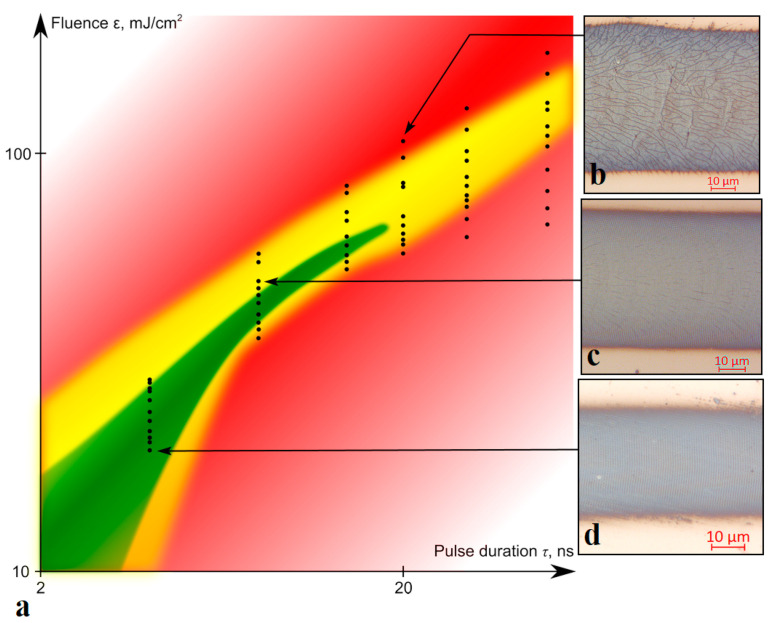
(**a**) Experimentally determined recording regimes for obtaining oxidative LIPSSs on titanium films of the selected thickness: the green area is homogeneous, with contrast structures over the entire track area; the yellow area has structures predominantly along the edges of the tracks; the red area has no structures or film cracking over the entire track area. Black dots mark the experimentally obtained results. Recording regimes (with the corresponding points marked in (**a**)): (**b**) *τ* = 20 ns, *P_av_* = 100 mW, *f* = 15 kHz, *ε* = 100 mJ/cm^2^; (**c**) *τ* = 8 ns, *P_av_* = 100 mW, *f* = 30 kHz, *ε* = 48 mJ/cm^2^; (**d**) *τ* = 4 ns, *P_av_* = 100 mW, *f* = 60 kHz, *ε* = 23 mJ/cm^2^.

**Figure 5:**
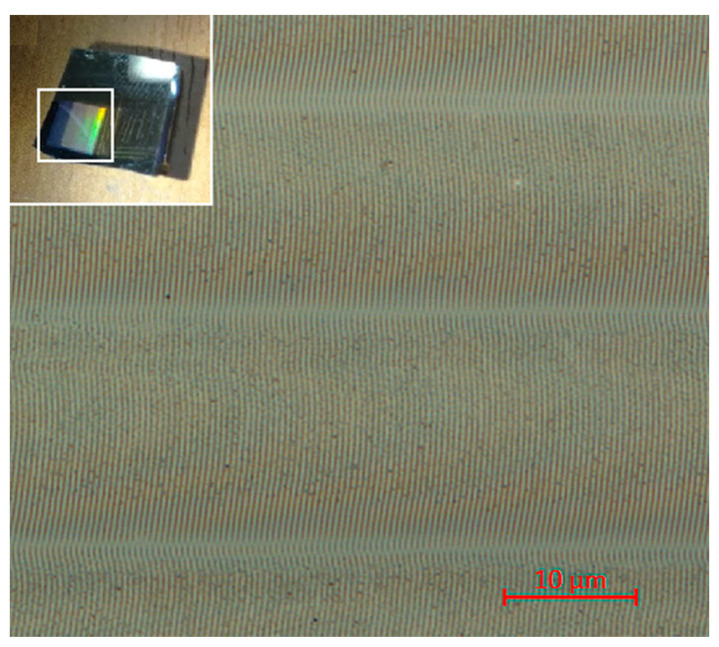
Fragment of a structure consisting of sequentially recorded tracks with LIPSSs using an initial partially polarized laser beam. Recording regime: *τ* = 4 ns, *P_av_* = 90 mW, *f* = 50 kHz, *ε* = 20 mJ/cm^2^ (for a single track see Figure 2a). Sample photo is shown in the inset.

The experimentally defined regimes favorable for the formation the oxidative LIPSSs on the thin metallic titanium films are shown in Figure 4a. With an increase in the duration or fluence of the pulses, a shift of the LIPSSs from the center of the irradiated region to the zone with a lower radiation intensity was observed (see Figure 2c and Figure 4c), until their complete disappearance. The optimal repetition rate of nanosecond pulses lies in the range from 40 to 70 kHz (Figure 4d); however, this range expands with a reduction in the duration of a single pulse. Thus, contrast, ordered, and regular LIPSSs were observed when exposed to pulses with duration of 4 ns in a wide range of their repetition rates from 30 to 100 kHz. Observed restrictions on the pulse repetition rates are most likely to be explained by their connection with laser fluence. A decrease in the pulse repetition rate while maintaining the average power leads to an increase in the fluence and, consequently, an increase in the maximum peak value of the film temperature. This is followed by an increase of thermal-induced stresses, and, finally, the experimentally observed cracking of the film material and the disappearance of the regular LIPSSs (Figure 4b). An increase in the pulse repetition rate beyond the optimal range most likely leads to a decrease in the average fluence below the threshold values required for the LIPSS excitation. Reproduction of the LIPSS orientation as a result of positive feedback was also demonstrated by recording several tracks with a slight overlap (about 8%) (Figure 5). It can be noted that the direction, rotation angle, and location of the LIPSSs were reproduced on different tracks recorded sequentially and independently of each other. This reproducibility can be useful for functional structuring of the film surface under the considered conditions.

## 4. Conclusions

In the presented work, we demonstrated the formation of oxidative LIPSSs on titanium films under the action of nanosecond pulses. The mechanism of LIPSS formation under the considered conditions confirms the model proposed earlier in [12,14,22], which is based on the interference of the initial laser radiation, with that scattered by the initial film roughness. The results of AFM, optical microscopy, and subsequent 2D-FFT analysis show that the depth of the oxidative relief formed on the titanium film is stable, with an average of 90 nm, and its period is about 0.7 *λ*. It is significant that the formation of LIPSSs occurs in the preablative oxidative temperature regime at temperatures below the melting point, due to which the structures have high reproducibility and order (deviations in the value of the period do not exceed 0.02 μm). The LIPSS reproducibility is also facilitated by the recording method, in which the formation of the relief occurs under the influence of 24,000–80,000 nanosecond pulses (for 0.8 s) that successively irradiate the affected area. The action of subsequent pulses consolidates and significantly enhances the relief formed as a result of the scattering of the first pulses on the initial roughness of the film. This positive feedback contributes to the appearance of LIPSSs (under the influence of partially polarized radiation) with the predominant formation direction. The experimentally defined optimal regimes for the LIPSS formation were pulse durations in the range of 4–14 ns and fluences in the range of 10–70 mJ/cm^2^, while the range of the latter is limited by fluence values sufficient for the excitation of LIPSSs but not exceeding the films’ cracking thresholds. It was shown that since the pulse repetition rate is a technological factor that affects the energy density, it can be used to control the occurrence of LIPSSs (for example, under studied conditions, the frequency values suitable for the formation of LIPSS were 30–70 kHz at *τ* = 8 ns ceteris paribus). Yb-laser as part of the MiniMarker laser processing complex is a commercially-available, economically competitive system that allows obtaining high-quality uniform structures far more efficiently than by using a femtosecond laser system [12]. Controlling the direction of formation of the oxidative relief under the studied conditions can be achieved relatively easily, simply by changing the scanning direction of a partially polarized laser beam or by rotating the plane of polarization of radiation, which opens up new possibilities for applied surface functionalization.

## Figures and Tables

**Table 1:** Physical and optical characteristics of titanium and BK7 glass.

**Titanium [33,34]**	
Density, *p_1_*	4.5 × 10^3^ kg/m^3^
Thermal capacity, *c_1_*	530.8 J/(kg·K)
Absorbance coefficient *A_1_* at *λ* = 1.07 μm	0.42
**BK7 glass [35]**	
Density, *p_1_*	2.5 × 10^3^ kg/m^3^
Thermal capacity, *c_2_*	720 J/(kg·K)
Thermal diffusivity, *a_2_*	0.6 × 10^−6^ m^2^/s
